# An Economic Evaluation of Resistance Training and Aerobic Training versus Balance and Toning Exercises in Older Adults with Mild Cognitive Impairment

**DOI:** 10.1371/journal.pone.0063031

**Published:** 2013-05-14

**Authors:** Jennifer C. Davis, Stirling Bryan, Carlo A. Marra, Devika Sharma, Alison Chan, B. Lynn Beattie, Peter Graf, Teresa Liu-Ambrose

**Affiliations:** 1 Centre for Clinical Epidemiology and Evaluation, University of British Columbia, Vancouver, Canada; 2 Faculty of Pharmaceutical Sciences, University of British Columbia, Vancouver, Canada; 3 Department of Physical Therapy, University of British Columbia, Vancouver, Canada; 4 Brain Research Centre, University of British Columbia, Vancouver, Canada; University of Bath, United Kingdom

## Abstract

**Background:**

Mild cognitive impairment (MCI) represents a critical window to intervene against dementia. Exercise training is a promising intervention strategy, but the efficiency (i.e., relationship of costs and consequences) of such types of training remains unknown. Thus, we estimated the incremental cost-effectiveness of resistance training or aerobic training compared with balance and tone exercises in terms of changes in executive cognitive function among senior women with probable MCI.

**Methods:**

Economic evaluation conducted concurrently with a six-month three arm randomized controlled trial including eighty-six community dwelling women aged 70 to 80 years living in Vancouver, Canada. Participants received twice-weekly resistance training (n = 28), twice weekly aerobic training (n = 30) or twice-weekly balance and tone (control group) classes (n = 28) for 6 months. The primary outcome measure of the Exercise for Cognition and Everyday Living (EXCEL) study assessed executive cognitive function, a test of selective attention and conflict resolution (i.e., Stroop Test). We collected healthcare resource utilization costs over six months.

**Results:**

Based on the bootstrapped estimates from our base case analysis, we found that both the aerobic training and resistance training interventions were less costly than twice weekly balance and tone classes. Compared with the balance and tone group, the resistance-training group had significantly improved performance on the Stroop Test (*p* = 0.04).

**Conclusions:**

Resistance training and aerobic training result in health care cost saving and are more effective than balance and tone classes after only 6 months of intervention. Resistance training is a promising strategy to alter the trajectory of cognitive decline in seniors with MCI.

**Trial Registration:**

ClinicalTrials.gov NCT00958867.

## Introduction

Cognitive decline is one of the most prominent health care issues of the 21st century. Worldwide, over 35 million people have dementia and one new case is detected every four seconds [1]. The number of people affected is projected to be over 80 million by 2040 [1]. In Canada, over 500 000 individuals have dementia today; over one million individuals are expected to have dementia by 2038 [2]. The resultant burden imposed on long term care facilities is projected to increase 10-fold [2]. Given the ageing population and the number of individuals affected by cognitive decline, the societal value of cost-effective intervention strategies is critically important in an environment where health care resources are scarce and finite [3].

Indeed, if the onset and progression of dementia were delayed by a modest one year, it would lead to 9 million fewer cases of disease in 2050 [3]. In Canada, the total economic burden attributable to dementia was estimated at approximately 15 billion dollars accounting for direct and indirect costs [2]. Direct health care costs may include items such as health care professional visits, admissions to hospital, laboratory tests or investigations and medications. Indirect costs include items such as family members time spent helping the patient or loss of work productivity. By 2038, the projected economic burden attributable to dementia is 872 billions dollars; 570 billion are direct health care costs and 302 billion are indirect costs representing the additional burden to society [2].

Cognitive decline results in significant burden placed on our health care system. Specifically, multiple studies have demonstrated that dementia is associated with increased health care resource utilization. Specifically, individuals with dementia accrue three times the yearly out of pocket expenses compared with non-demented individuals [4]. Out of pocket expenses may include medications not covered by health care insurance, caregiver time and transportation.

Dementia slowly limits an individual’s ability to function independently. As such, individuals with dementia who live long enough are will use long-term care facilities such as nursing homes – all of which have marked direct and out of pocket expenses [4]–[6]. It is projected that by 2020, Canada will have well over 10 million seniors with moderate to severe functional dependence [7]. Functional dependence was the most significant contributor to an annual cost of dementia that had already reached $4 billion in the 1990s [8].

Current evidence emphasizes the importance of behavioural prevention strategies for cognitive decline such as physical activity [9]–[14]. As previously highlighted by Erickson and Kramer [15], physical activity provides clear benefits for cognition among seniors. These neuroscientists contend that “physical activity is an inexpensive treatment that could have substantial preventative and restorative properties for cognitive and brain function” [16]. Randomized trials of various exercise interventions have demonstrated that exercise has benefits for balance, cognition, body composition, and cardiovascular health [17]–[23]. In summary, evidence indicates more broadly that targeted physical activity interventions are associated with improved cognitive function among older adults [9].

We previously demonstrated that two doses, once or twice weekly resistance training compared with balance and tone classes significantly reduce health care resource utilization among older adults [24]. These cost-saving benefits were further sustained 12 months after the intervention ceased [25], [26]. Hence, it is reasonable to speculate that targeted interventions that promote mobility may indeed save health care dollars.

Mild cognitive impairment (MCI) is a well-recognized risk factor for both Alzheimer’s Disease [27] and functional dependence [28], [29] which are both associated with decline in health related quality of life (HRQoL). One recent study demonstrated that significant declines in quality of life are notable in individuals with MCI. These declines in HRQoL were associated with neuropsychiatric and function decline. This may highlight that there is an ideal window of opportunity to intervene with physical activity interventions among older adults to promote maintenance and improvements in HRQoL that contribute to healthy aging and that reduce the overall economic burden on the health care system.

Previously, we demonstrated that among 70–80 year old community-dwelling women with subjective memory complaints, six months of twice-weekly resistance training (RT) improved selective attention and conflict resolution, associative memory, and regional patterns of functional brain plasticity, compared with twice-weekly balance and toning exercises [30]. In contrast, six months of twice-weekly aerobic training (AT) significantly improved physical function [30]. This study was the first to assess the efficacy of both resistance training and aerobic training on cognitive performance, functional plasticity, and physical function in senior women with probable MCI [30].

What remains unknown is whether resistance training or aerobic training compared with balance and toning exercises in community dwelling older women will reduce total healthcare resource utilization while improving cognitive function and thus provide better value for money. Therefore, we designed a concurrent, prospective economic analysis using individual level data on cost and effectiveness outcomes as part of the Exercise for Cognition and Everyday Living (EXCEL) study, a three arm randomized controlled trial [30]. The main outcome results are reported elsewhere [30].

Our primary objective for the economic evaluation was to determine the incremental cost effectiveness ratio (cost per seconds gained or lost on Stroop Test) of twice weekly resistance training or twice weekly aerobic training compared with twice weekly balance and tone classes (BAT). We modeled the BAT program on a popular provincial-wide exercise program designed to reduce falls among seniors with low bone mass (the Osteofit program). The BAT control group is representative of exercise programs commonly available in the community such as Osteofit, yoga or Tai Chi but it is not specifically designed to combat cognitive decline. We did not use a ‘usual practice’ arm in this analysis because it would not reflect current practice in the community. For communities where such classes are not offered our approach may provide an overly conservative estimate of health benefit.

## Materials and Methods

Ethical approval was obtained from the Vancouver Coastal Health Research Institute and the University of British Columbia’s Clinical Research Ethics Board. All participants provided written informed consent.

### Overview of Economic Evaluation

We used a Canadian healthcare system perspective in our cost effectiveness analysis and a six-month time horizon. We analyzed the data from the EXCEL trial on an intention to treat basis. The main outcome for our primary (cost effectiveness) analysis was the incremental cost per seconds gained or lost on Stroop Test.

We previously reported study design, study sample, participant recruitment, randomization, demographics, methods and primary outcome results of the EXCEL trial [30]. Briefly, the study sample included 86 community dwelling women aged 70 to 80 years. Cognitive response to exercise differs between the sexes; therefore, our sample consisted solely of women [31].

### Sample Size

The required sample size for this study was calculated based on predictions of six-month changes in the Stroop Test. We specifically predicted 12% improvement for both RT and AT and 10% deterioration in the BAT group (i.e., control group). These estimates are based on our previous work in seniors aged 70 years and older with a significant history of falls [32] and healthy community-dwelling senior women [9]. Assuming a 15% attrition rate and using an alpha level of <0.05, 29 participants per group ensured a power of 0.80.

Participants enrolled in the EXCEL study had a Mini Mental State Examination (MMSE) score ≥24 (i.e., were cognitively intact), answered “yes” to the question “Do you have any difficulty with your memory?” [33], scored ≥6/8 on the Lawton and Brody [34] Instrumental Activities of Daily Living, obtained their physician’s clearance to start a supervised exercise program, and visual acuity 20/40 or better with or without corrective lenses. The secondary objective of EXCEL that related to our economic evaluation was to determine whether twice weekly resistance training or twice weekly aerobic training provided better value for money compared with twice-weekly balance and tone classes.

### Overview of Intervention and Control Groups

The interventions for the EXCEL study included three participant groups: twice weekly resistance training, twice weekly aerobic training and the BAT group, twice weekly balance and tone classes. All classes, offered two times per week over 6-months, were 60 minutes long, with a 10-minute warm-up, 40 minutes of core content, and a 10-minute cool down period.

#### Resistance Training

The RT program used a progressive, high intensity protocol. The protocol for the RT program has been described previously [9]. Both a Keiser® Pressurized Air system and free weights were used to provide the training stimulus. The Keiser-based exercises consisted of biceps curls, triceps extension, seated row, latissmus dorsi pull downs, leg press, hamstring curls, and calf raises. The intensity of the training stimulus was at a work range of six to eight repetitions (two sets). The training stimulus was subsequently increased using the 7RM method – when two sets of six to eight repetitions were completed with proper form and without discomfort. Other key strength exercises included mini-squats, mini-lunges, and lunge walks. The number of sets completed and the load lifted for each exercise was recorded for each participant at every class.

#### Aerobic Training

The AT program was an outdoor walking program. The intensity of the training stimulus was at approximately 40% of one’s age specific target heart rate (i.e., heart rate reserve; HRR) and systematically progressed 60% of HRR over a five-month period. Once the target of 60% of HRR was achieved, it was sustained by the participant for the remainder of the intervention period. We used both objective and subjective methods to monitor exercise intensity. Objectively, exercise intensity was monitored through heart rate monitors. Subjectively, participants monitored the intensity of their workouts by the Borg’s Rating of Perceived Exertion [35] and the “talk” test [36], [37]. For the “talk” test, participants were instructed to to initially walk at a pace where they conversed comfortably without effort and progress to a walking pace where conversation required effort.

#### Balance and Tone

The protocol for the BAT program has been described previously [9]. The BAT program consisted of stretching exercises, range of motion exercises, basic core-strength exercises including kegals (i.e., exercises to strengthen the pelvic floor muscles), balance exercises, and relaxation techniques. Other than bodyweight, no additional loading (e.g., hand weights, resistance bands, etc.) was applied to any of the exercises. There is no evidence that these exercises improve cognitive function [38]. This group served to control for confounding variables such as physical training received by traveling to the training centres, social interaction, and changes in lifestyle secondary to study participation.

Compliance, expressed as the percentage of the total classes attended, was calculated from these attendance sheets.

### Costs

We used a patient self-complete questionnaire to track total healthcare resource utilization prospectively for each participant for 6 months. We collected these questionnaires at 3-month time intervals during the 6-month time horizon where possible. For missing data at the 3-month time interval, we asked participants to complete the questionnaire over a 6-month time horizon. The major resource categories were: visits to healthcare professionals (including general practitioners, specialists, physiotherapists etc); all visits, admissions or procedures carried out in a hospital; and laboratory and diagnostic tests. We calculated the costs of delivering the RT, AT and BAT programs for 6 months. Our base case analysis considered the costs of delivering the program and all healthcare resource use. We excluded research protocol driven costs from our analysis as these do not reflect the cost of implementation in a real world setting. However, the costs of delivering the program included staff time (fitness trainer time), room use, equipment provided as part of the facility and any building overhead costs.

For each component of health resource utilization, we assigned a unit cost. All costs for admission to hospital were based on the fully allocated cost model of a tertiary care hospital, Vancouver General Hospital. For unit costs of healthcare professionals, we based costs on fee for service rates from the British Columbia Medical Services Plan 2009 price list. Unit costs for specialized services such as physiotherapy, chiropractic or naturopathic medicine were taken from the BC Association website for each specialty. We did not have access to the actual cost of each item of health resource utilization and therefore assigned a unit cost specific to the health professional seen, procedure or laboratory test performed. We inflated costs to 2011 Canadian dollars using the consumer price index reported by Statistics Canada. Discounting was not relevant given our analytic time horizon.

### Effectiveness Outcomes

#### Primary outcome measure

This study focused on three executive cognitive functions: selective attention and conflict resolution, set shifting, and working memory. Our primary outcome measure was the specific executive cognitive function of selective attention and conflict resolution, as measured by a paper version of the Graf, Uttl, and Tuokko’s [39] Stroop Test [40] to assess selective attention and conflict resolution. We previously demonstrated that it responds to exercise training [9], [32] and used those observed changes for our sample size calculation.

For the Stroop Test, there were three conditions. Each condition included 80 items, arranged in four columns of 20 items. First, participants were instructed to read out words printed in black ink (e.g., BLUE). Second, they were instructed to read out the colour of coloured-X’s. Four colours were used – red, blue, green, and yellow. Finally, they were shown a page with colour-words printed in incongruent coloured inks (e.g., the word “BLUE” printed in red ink). Participants were asked to name the ink colour in which the words are printed (while ignoring the word itself). There were 80 trials for each condition and we recorded the time participants took to read each condition. The ability to selectively attend and control response output was calculated as the time difference between the third condition and the second condition. This is a standard difference score used to index selective attention and conflict resolution [41]. Smaller time differences indicate better performance.

For the economic evaluation, to evaluate the incremental benefit of the intervention, we estimated the incremental Stroop Interference score for each participant. First, we determined the Stroop Interference change score by calculating the time difference between the third condition and the second condition at baseline minus trial completion for each group. Second, to ascertain the incremental Stroop Interference score, we calculated the mean difference between the above calculated change scores between the RT and BAT groups and the AT and BAT groups. Greater positive change scores over time indicate greater improvement over time. All statistical analyses were carried out using STATA version 11.0.

### Cost Effectiveness Analysis

We calculated the cost and effectiveness data with the complete data set. We calculated the incremental cost effectiveness ratio for both AT and RT compared with BAT. We used nested imputation and nonparametric bootstrapping to model uncertainty around the estimates for costs and effectiveness. For each of the five cycles, we bootstrapped the complete dataset. For each cycle of bootstrapping, we calculated the total healthcare resource use cost and Stroop Interference change score by group allocation. We evaluated the contribution of each cost item in relation to the total healthcare resource use estimated for each group. We used scatterplots on the cost effectiveness plane based on 5000 iterations of bootstrapping to estimate the joint distribution of cost and effectiveness outcomes [42].

## Results

We present baseline study characteristics in Table 1. The mean age of participants was 75 years. The average mean Functional Comorbities Index score was 3 and the average Montreal Cognitive Assessment (MOCA) score was 22 confirming this is a MCI population. The minimum MOCA score overall was 14 and the maximum was 27. For the BAT group, the minimum MOCA score overall was 16 and the maximum was 27, for the AT group the minimum MOCA score overall was 14 and the maximum was 26 and for the RT group, the minimum MOCA score overall was 15 and the maximum was 26. There were no drop outs from the BAT group, six drop outs from the AT group and 2 drop outs from the RT group.

**Table 1:** Characteristics of participants at entry to trial.

Characteristic	Twice weekly balance and tone (n = 28)	Twice weekly aerobic training (n = 30)	Twice weekly resistance training (n = 28)
	Mean (SD) or Frequency (%)	Mean (SD) or Frequency (%)	Mean (SD) or Frequency (%)
Age, years	75.0 (3.7)	75.5 (3.5)	74.1 (3.6)
Weight, kg	66.4 (13.9)	65.0 (12.6)	65.4 (10.4)
Height, cm	158.2 (7.3)	159.4 (6.0)	159.1 (7.0)
Hip girth, cm	87.9 (12.0)	87.9 (12.1)	87.6 (12.6)
Waist girth, cm	103.2 (13.1)	100.6 (12.5)	101.9 (10.6)
Waist to hip ratio	0.8 (0.1)	0.8 (0.1)	0.9 (0.2)
PPA score	1.5 (1.2)	1.5 (0.7)	1.4 (1.0)
MoCA (max 30 points)	22.5 (2.8)	22.2 (2.8)	21.4 (3.4)
Functional Comorbidity Index score	2.6 (2.2)	2.9 (1.5)	3.0 (1.8)
Arthritis	12 (46)	18 (60)	16 (52)
Osteoporosis	11 (42)	15 (50)	8 (26)
Asthma	2 (8)	5 (17)	4 (13)
Chronic Obstructive Pulmonary Disease	1 (4)	2 (7)	1 (3)
Angina	2 (8)	1 (3)	1 (3)
Congestive heart failure	0 (0)	1 (3)	0 (0)
Heart attack	2 (8)	0 (0)	1 (3)
Neurological disease	0 (0)	0 (0)	1 (3)
Stroke or TIA	2 (8)	1 (3)	1 (3)
Peripheral vascular disease	1 (4)	2 (3)	2 (6)
Diabetes type I and II	1 (4)	4 (13)	4 (13)
Upper gastrointestinal disease	5 (19)	8 (27)	12 (39)
Depression	0 (0)	0 (0)	3 (10)
Anxiety or panic disorders	3 (12)	3 (10)	4 (13)
Visual impairment	8 (31)	10 (33)	11 (35)
Hearing impairment	1 (4)	1 (3)	4 (13)
Degenerative disc disease (back disease, spinalstenosis or severe chronic back pain)	5 (19)	4 (13)	4 (13)
Obesity	0 (0)	0 (0)	0 (0)

### Healthcare Use and Costs

**Figure 1:**
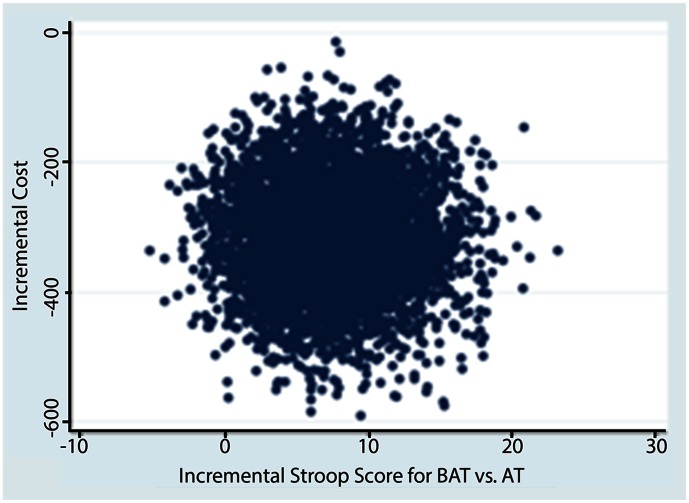
Cost effectiveness plane depicting scatterplot of bootstrapped estimates of incremental cost and effectiveness for comparison between AT and BAT.

**Figure 2:**
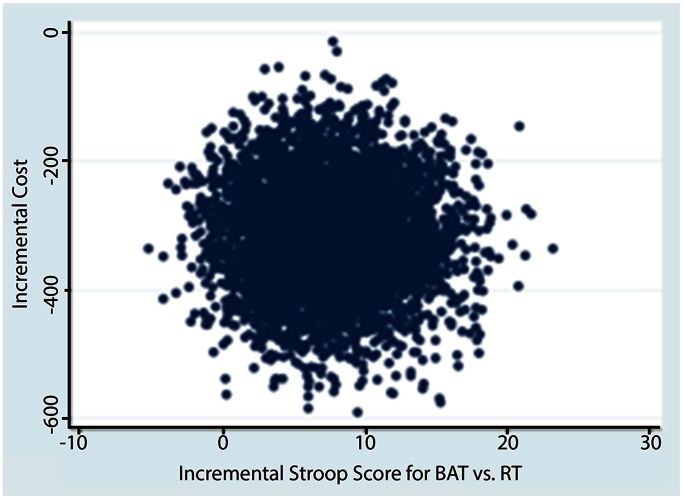
Cost effectiveness plane depicting scatterplot of bootstrapped estimates of incremental cost and effectiveness for comparison between RT and BAT.

Of individuals who completed this 6-month randomized controlled trial, complete healthcare resource utilization data were provided by all participants. Unit costs for healthcare cost items are provided in Table 2. The mean total healthcare costs were notably lower in the cost-saving region of the cost-effectiveness plane for the AT and RT groups compared with BAT (p<0.05) (see Table 3 and Figures 1 and 2). Of note, a key driver of total health care resource utilization costs were visits to health care professionals (Table 3). Specifically, we note that the mean costs for health care profession visits and hospital admissions were larger for the RT group compared with the AT group.

**Table 2:** Unit costs for each component of resource utilization.

Item	Value 2010 CAD$Ý	Unit	Reference
Cost of delivering twice weekly balance and tone classes	353.06	Cost per person year	Study records
Cost of delivering twice weekly aerobic training	352.15	Cost per person year	Study records
Cost of delivering twice weekly resistance training	353.06	Cost per person year	Study records
Health care professional visit, mean (standard deviation)	566 (593)	Cost per visit	2010 Medical services plan
Admissions to hospital	97 (198)	Cost per day	2005 Vancouver General Hospital fully allocated cost model*
Laboratory procedures, mean (standard deviation)	63 (94)	Cost per procedure	2009 Medical services plan

Ý Canadian dollars (CAD) at 2010 prices.

* Taken from the fully allocated cost model at Vancouver General Hospital.

**Table 3:** Results of base case analysis.

	Twice weekly balanceand tone (n = 28)	Twice weekly aerobictraining (n = 30)	Twice weekly resistancetraining (n = 28)
Cost of delivering program per person (2010 CAD $^¶^)	353.06	352.15	353.06
Mean (SD) total healthcare resource use cost (2010 CAD $)	1179 (934)	863 (448)	1146 (636)
Mean (SD) total health care professional related costs (2010 CAD $)	682 (811)	426 (380)	592 (499)
Mean (SD) hospital admission related costs (2010 CAD $)	82 (183)	40 (95)	151 (254)
Mean (SD) laboratory tests or investigations costs (2010 CAD $)	91 (113)	52 (92)	46 (72)
Mean incremental costs for total healthcare resource use (2010 CAD $)	reference	−316	−33
Stroop* at Baseline	55.98 (25.2)	57.11 (42.1)	52.22 (26.7)
Stroop* at Final	54.69 (31.3)	48.27 (31.3)	44.61 (25.8)
Mean change in Stroop* (Baseline – Final)	1.37 (15.26)	8.83 (41.86)	9.13 (19.88)
Incremental cost per incremental mean change in Stroop* based on:	reference||	7.5	7.8
Total healthcare resource use costs^Y˙^	reference||	dominates§ Figure 1	dominates§ Figure 2

* Stroop CW – Stroop C.

§ For these strategies it was not appropriate to calculate an incremental cost effectiveness ratio because the intervention strategy were less costly and more effective than the balance and tone group (i.e., the intervention was less costly and more effective than the balance and tone group in each of these cases).

|| Reference indicates that the balance and tone group is the group from with the AT and RT intervention groups are compared.

### Exercise Adherence

The exercise compliance over the 26-week trial was 57% (16.1). The RT group had an average compliance of 54% (14.7), 60% (18.7) for the AT group, and 59% (14.8) for the BAT group.

### Effectiveness Outcomes

The mean change in Stroop Interference time from baseline to final was 1.4 (15.3) for BAT, 8.8 (41.9) for AT and 9.1 (19.9) for RT group. The incremental Stroop Interference time for AT compared with BAT was 7.5 seconds and for RT compared with BAT was 7.8 seconds.

### Cost Effectiveness Analysis

Based on the bootstrapped estimates from the cost-effectiveness plane (Figures 1 and 2) of our base case analysis we found that AT and RT were less costly and more effective than BAT. We did not report cost effectiveness acceptability curves here because both interventions were cost-saving and the resistance and aerobic training intervention were more effective.

## Discussion

### Key Findings

From the Canadian healthcare system perspective, the incremental cost per incremental Stroop change score indicated that both aerobic training and resistance training resulted in lower healthcare costs and were more effective than twice weekly balance and tone classes – both aerobic training and resistance training provides better value for money than twice weekly balance and tone classes. Further, the mean incremental Stroop interference scores for both the aerobic training and resistance training groups were greater than five seconds. From the normative data published from the Masstricht Aging Study [43], a 5-second interval represents the difference in interference among women with average to high level of education between the mean ages of 65, 70, and 75 years (i.e., a clinical important change). To our knowledge, there is no published data indicating what a 5-second improvement in the Stroop Interference score represents in terms of delaying the onset of dementia or institutionalization. Further, the Stroop Interference time is significantly associated with dementia risk [44]. We highlight that determining a clinically meaningful second improvement in Stroop Interference score is an essential research priority moving forward. For the cost analyses, we found that key drivers for health resource utilization were visits to health care professionals and admissions to hospital.

In explaining our findings, we highlight that economic evaluations do not rely on a statistically significant difference in effectiveness and costs between the intervention and the control group to drive the conclusion [45]. The key factor is the mean point estimate and the location of the greatest proportion of mean point estimates on the cost-effectiveness plane that are generated from the bootstrapped replications. The point estimates are plotted on the four quadrants of the cost-effectiveness plane (e.g. Figures 1 and 2) [45]. Exploring the uncertainty in economic evaluations uses the Bayesian perspective rather than a Frequentist perspective. Thus, rather than testing statistical significance, we explored uncertainty using bootstrap methods. Figures 1 and 2 demonstrate that for RT or AT compared with BAT, 100% of the bootstrapped cycles were located in the southeast quadrant. This indicates that resistance training is more effective and less costly than the BAT classes.

Critical to this novel study population, recent RCTs provide preliminary evidence that the benefits of two types of exercise – aerobic and resistance training – extend to older adults with MCI [33], [46], [47]. Although previous research has demonstrated that once or twice weekly resistance training is an effective [48] and cost-effective [49] strategy to combat cognitive decline among community dwelling older adults, such data do not exist for individuals with MCI. Mild cognitive impairment is a well recognized risk factor for dementia ^3^ and impaired mobility [50]. As such, targeting such interventions at individuals with MCI may provide a critical window of opportunity for intervening and cost-effectively altering the trajectory of functional decline in older adults. However, to date there is a dearth of economic evaluations in this field.

### Comparison with Other Studies

Our primary finding concurs with and extends our previous work in health community dwelling older women [24]. Previously we demonstrated that 12 months of once or twice weekly resistance training provided better value for money (i.e., lower costs and improved health related quality of life) than balance and tone classes among cognitively healthy community dwelling women aged 65–75 years old [24]. In the current study, we found the resistance training group had significantly improved cognitive function (i.e., performance on the Stroop Test (*p* = 0.04) and the aerobic training group significantly improved general balance and mobility (*p* = 0.03) and cardiovascular capacity (*p* = 0.04) after only 6 months [9]. Further, both the resistance training and aerobic training groups had lower health care resource utilization costs. Thus, we provide novel preliminary evidence that resistance training and aerobic training are effective and economically attractive solutions that significant and multiple benefits this high risk population.

### Limitations

In considering the limitations of our study, we highlight that a gold standard economic evaluation conducted alongside a clinical trial will include the following four characteristics: 1) a control group commonly used in standard practice, 2) adequate power, 3) sufficient followup time to assess full health gains or losses and 4) appropriate time frame to aid in decision making and adoption [51]. The BAT group used in our study is not part of usual care practice. However, it is consistent with programs commonly available that are not primarily aimed at reducing cognitive decline. As such, our economic evaluation may provide an overly conservative estimate of the health benefit of the AT or RT classes given that participants may also have experienced a positive health benefit from the balance and tone classes. The EXCEL study was a proof of concept study that was powered for the primary clinical outcome – the Stroop Test. This study was not powered for the economic evaluation primary outcome and thus the sample size may be too small to detect robust differences. The time horizon of our study was limited to the 6-month study duration. Previous research demonstrates that resistance training in older adults has long term health benefits that would ideally be captured by a longer time horizon [52]. Further, we note that participants self-reported health resource utilization via questionnaire. Given that individuals in this study had probable MCI, we highlight that the absolute value of health resource utilization costs may be underestimated. Assuming randomization was successful, we anticipate minimal impact on the calculated incremental costs for the economic evaluation. Lastly, we did not model lifetime estimates for costs and cognitive outcomes in this study and we do not have sufficient data to report long term (e.g. lifetime) costs and consequences of this intervention.

### Strengths

The strength of this economic evaluation conducted alongside a clinical trial is that we collected information on cost and effectiveness outcomes prospectively and thus minimized recall and response bias. Further, we collected healthcare resource utilization every 3 months to minimize recall bias. To our knowledge, this is the first economic evaluation to examine the best value for money of two different well recognized and effective exercise training approaches (aerobic versus resistance) [9], [31], [53] aimed at reducing cognitive decline among a population older adults with MCI.

### Conclusions and Future Directions

In conclusion, resistance training and aerobic training are promising strategies to alter the trajectory of cognitive decline in seniors with MCI [30]. We now know that both resistance training and aerobic training result in health care cost saving and are more effective than balance and tone classes after only 6 months of intervention. Future research that includes evaluating these interventions over a longer (lifetime) time horizon and among a larger sample is needed to confirm these preliminary findings.

## Supporting Information

Table S1 **Health resource utilization questionnaire.**
(DOC)
