# Supplementary material for: An Economic Evaluation of Resistance Training and Aerobic Training versus Balance and Toning Exercises in Older Adults with Mild Cognitive Impairment
Source: PLoS One. 2013 May 14;8(5):e63031. doi: 10.1371/journal.pone.0063031 (PMC3653911; doi:10.1371/journal.pone.0063031)
Supplement: Table S1 — Health resource utilization questionnaire. (DOC) [file pone.0063031.s001.doc]

APPENDIX

| **HEALTH RESOURCE UTILIZATION**  Followup Time point:  Date: _______________  Participant Label   | This questionnaire asks about your usage of the health care system since **MIDPOINT**. We want to know what health care professionals you have seen and what costs, if any, you may have had. We know there are costs related to your health that are often hidden or not documented.  **1. Office visits to health professionals (of any type) since MIDPOINT:**  Please report below about any visits you have made to health professionals since **MIDPOINT**. Some of the people you may have seen include your family physician, medical specialists, physiotherapists, chiropractors, acupuncturists, nurse practitioners, dentists, etc. If you have visited any health professional more than once, please tell me about each visit separately. If your visit was related to a fall please check the fall related check box.  🞏 **Check here if no visits to any health professional since MIDPOINT *(Skip to question 2)*** | | --- |  | **a.** Health Professional:  ____________ | Date of visit:  __________ | Reason for visit:  ___________________________ | | --- | --- | --- | | 🞏 Fall related | 🞏 No Therapy | Most important therapy prescribed/given:  ___________________________ | | 🞏 No Procedure | Most important procedure/service that performed:  ___________________________ | | **b.** Health Professional:  ____________ | Date of visit:  __________ | Reason for visit:  ___________________________ | | 🞏 Fall related | 🞏 No Therapy | Most important therapy prescribed/given:  ___________________________ | | 🞏 No Procedure | Most important procedure/service that performed:  ___________________________ | | **c.** Health Professional:  ____________ | Date of visit:  __________ | Reason for visit:  ___________________________ | | EXCEL-HRU Page 1 of 5 T3  🞏 Fall related | 🞏 No Therapy | Most important therapy prescribed/given:  ___________________________ | | 🞏 No Procedure | Most important procedure/service that performed:  ___________________________ | |
| --- | --- | --- | --- | --- | --- | --- | --- | --- | --- | --- | --- | --- | --- | --- | --- | --- | --- | --- | --- | --- | --- | --- | --- | --- | --- |
| EXCEL-HRU Page 2 of 5 T3  **HEALTH RESOURCE UTILIZATION**   | **d.** Health Professional:  ____________ | Date of visit:  __________ | Reason for visit:  ___________________________ | | --- | --- | --- | | 🞏 Fall related | 🞏 No Therapy | Most important therapy prescribed/given:  ___________________________ | | 🞏 No Procedure | Most important procedure/service that performed:  ___________________________ | | **e.** Health Professional:  ____________ | Date of visit:  __________ | Reason for visit:  ___________________________ | | 🞏 Fall related | 🞏 No Therapy | Most important therapy prescribed/given:  ___________________________ | | 🞏 No Procedure | Most important procedure/service that performed:  ___________________________ | | **f.** Health Professional:  ____________ | Date of visit:  __________ | Reason for visit:  ___________________________ | | 🞏 Fall related | 🞏 No Therapy | Most important therapy prescribed/given:  ___________________________ | | 🞏 No Procedure | Most important procedure/service that performed:  ___________________________ | | **g.** Health Professional:  ____________ | Date of visit:  __________ | Reason for visit:  ___________________________ | | 🞏 Fall related | 🞏 No Therapy | Most important therapy prescribed/given:  ___________________________ | | 🞏 No Procedure | Most important procedure/service that performed:  ___________________________ | | **h.** Health Professional:  ____________ | Date of visit:  __________ | Reason for visit:  ___________________________ | | 🞏 Fall related | 🞏 No Therapy | Most important therapy prescribed/given:  ___________________________ | | 🞏 No Procedure | Most important procedure/service that performed:  ___________________________ | |
| **HEALTH RESOURCE UTILIZATION**  EXCEL-HRU Page 3 of 5 T3   | **2. Visits to hospitals since MIDPOINT:**  Please tell me about any visits, admissions, tests or procedures you had in a hospital department  since **MIDPOINT.** Departments you may have visited include Radiology, Emergency, Day  Surgery, Cardiology, Intensive Care Unit, General Ward, etc.  🞏 **Check here if no visits to any hospital department since MIDPOINT *(Skip to question 3)*** | | --- |  | **a.** Type of hospital department:  ________________ | | Date of visit:  _________ | Reason for visit:  ________________________ | | --- | --- | --- | --- | | 🞏 Fall related | 🞏 No Tests/Procedures  Length of Stay:  ____________ (hrs / days) | | Most important tests/procedures performed:  ________________________  ________________________ | | **b.** Type of hospital department:  ________________ | | Date of visit:  _________ | Reason for visit:  ________________________ | | 🞏 Fall related | 🞏 No Tests/Procedures  Length of Stay:  ____________ (hrs / days) | | Most important tests/procedures performed:  ________________________  ________________________ | | **c.** Type of hospital department:  ________________ | | Date of visit:  _________ | Reason for visit:  ________________________ | | 🞏 Fall related | 🞏 No Tests/Procedures  Length of Stay:  ____________ (hrs / days) | | Most important tests/procedures performed:  ________________________  ________________________ | | **d.** Type of hospital department:  ________________ | | Date of visit:  _________ | Reason for visit:  ________________________ | | 🞏 Fall related | 🞏 No Tests/Procedures  Length of Stay:  ____________ (hrs / days) | | Most important tests/procedures performed:  ________________________  ________________________ | |
| **HEALTH RESOURCE UTILIZATION**   | **3. Tests or investigations since MIDPOINT:**  Please tell me about any tests or investigations you had (e.g. x-rays, ultrasounds, stress tests, mammograms) since **MIDPOINT.**  🞏 **Check here if no tests or investigations since MIDPOINT *(Skip to question 4)*** | | --- |  | **a.** Type of test/investigation:  ________________ | | Date of test/investigation:  _________________ | Site of test/investigation:  🞏 Hospital ( _________ )  🞏 Physician’s Office  🞏 Laboratory or x-ray site  (other than hospital)  🞏 Home | | --- | --- | --- | --- | | 🞏 Fall related | # of times in the past month: _______  Visit duration (days / hours): _______ | | | **b.** Type of test/investigation:  ________________ | | Date of test/investigation:  _________________ | Site of test/investigation:  🞏 Hospital ( _________ )  🞏 Physician’s Office  🞏 Laboratory or x-ray site  (other than hospital)  🞏 Home | | 🞏 Fall related | # of times in the past month: _______  Visit duration (days / hours): _______ | | | **c.** Type of test/investigation:  ________________ | | Date of test/investigation:  _________________ | Site of test/investigation:  🞏 Hospital ( _________ )  🞏 Physician’s Office  🞏 Laboratory or x-ray site  (other than hospital)  🞏 Home | | 🞏 Fall related | # of times in the past month: _______  Visit duration (days / hours): _______ | | | **d.** Type of test/investigation:  ________________ | | Date of test/investigation:  _________________ | Site of test/investigation:  🞏 Hospital ( _________ )  🞏 Physician’s Office  🞏 Laboratory or x-ray site  (other than hospital)  🞏 Home | | 🞏 Fall related | # of times in the past month: _______  Visit duration (days / hours): _______ | |   EXCEL-HRU Page 4 of 6 T3 |
| **HEALTH RESOURCE UTILIZATION**  EXCEL-HRU Page 5 of 5 T3   | **e.** Type of test/investigation:  ________________ | | Date of test/investigation:  _________________ | Site of test/investigation:  🞏 Hospital ( _________ )  🞏 Physician’s Office  🞏 Laboratory or x-ray site  (other than hospital)  🞏 Home | | --- | --- | --- | --- | | 🞏 Fall related | # of times in the past month: _______  Visit duration (days / hours): _______ | | | **f.** Type of test/investigation:  ________________ | | Date of test/investigation:  _________________ | Site of test/investigation:  🞏 Hospital ( _________ )  🞏 Physician’s Office  🞏 Laboratory or x-ray site  (other than hospital)  🞏 Home | | 🞏 Fall related | # of times in the past month: _______  Visit duration (days / hours): _______ | | | **g.** Type of test/investigation:  ________________ | | Date of test/investigation:  _________________ | Site of test/investigation:  🞏 Hospital ( _________ )  🞏 Physician’s Office  🞏 Laboratory or x-ray site  (other than hospital)  🞏 Home | | 🞏 Fall related | # of times in the past month: _______  Visit duration (days / hours): _______ | | | **h.** Type of test/investigation:  ________________ | | Date of test/investigation:  _________________ | Site of test/investigation:  🞏 Hospital ( _________ )  🞏 Physician’s Office  🞏 Laboratory or x-ray site  (other than hospital)  🞏 Home | | 🞏 Fall related | # of times in the past month: _______  Visit duration (days / hours): _______ | | | **i.** Type of test/investigation:  ________________ | | Date of test/investigation:  _________________ | Site of test/investigation:  🞏 Hospital ( _________ )  🞏 Physician’s Office  🞏 Laboratory or x-ray site  (other than hospital)  🞏 Home | | 🞏 Fall related | # of times in the past month: _______  Visit duration (days / hours): _______ | | |
